# Supplementary material for: Macroscopic helical chirality and self-motion of hierarchical self-assemblies induced by enantiomeric small molecules
Source: Nat Commun. 2018 Sep 18;9:3808. doi: 10.1038/s41467-018-06239-5 (PMC6143534; doi:10.1038/s41467-018-06239-5)
Supplement: Supplementary file 1 — Supplementary Information [file 41467_2018_6239_MOESM1_ESM.pdf]

Supplementary Information for

Macroscopic Helical Chirality and Self-motion of Hierarchical Self-assemblies Induced by Enantiomeric Small Molecules

Yang Yang[1, 2]†, Jie Liang[1]†, Fei Pan[1,3]†, Zhen Wang[1,2], Jianqi Zhang[1], Kamran Amin[1,2], Jin Fang[1], Wenjun Zou[1], Yuli Chen[3], Xinghua Shi[1, 2]* & Zhixiang Wei[1, 2]*

[1]CAS Key Laboratory of Nanosystem and Hierarchical Fabrication, CAS Center for Excellence in Nanoscience, National Center for Nanoscience and Technology, China, Beijing 100190, China.

[2]University of Chinese Academy of Sciences, Beijing 100049, China. [3] Institute of Solid Mechanics, Beihang University, Beijing 100191, China.

† These authors contributed equally to this work.

E-mail: shixh@nanoctr.cn; weizx@nanoctr.cn

Contents:

S1. Supplementary Figures.

S2. Supplementary Notes

Supplementary Note 1. Details about SAED.

Supplementary Note 2. Details about CD and *g*-factor.

Supplementary Note 3. Density functional theory calculation.

Supplementary Note 4. Multi-scale chemo-mechanical modeling and analysis.

Supplementary Note 5. Explanations for majority rules experiments.

Supplementary Note 6. Chirality perception mechanism of PANI:CSA stripes under stimuli of enantiomeric amines.

S1. Supplementary Figures

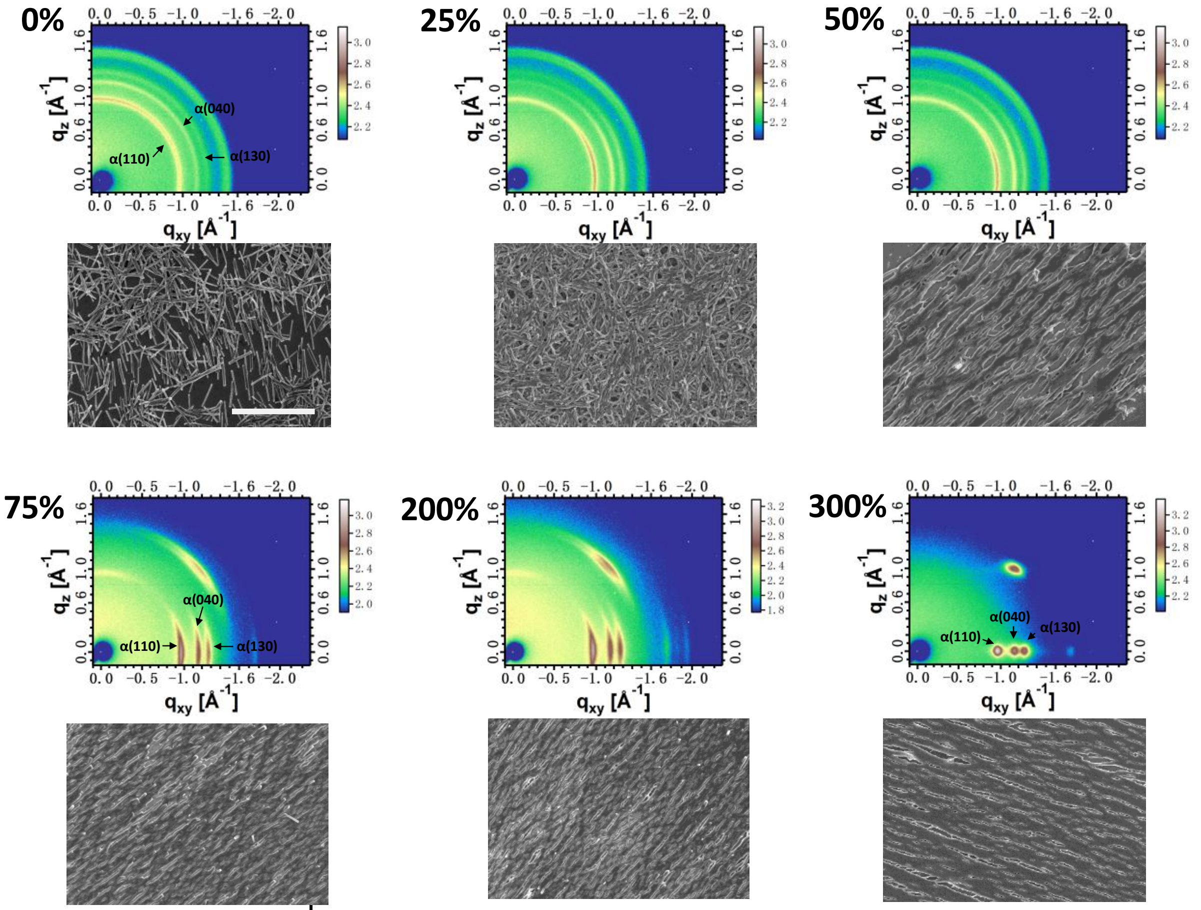

Supplementary Figure 1│Alignment in the polymer chain and its crystalline of the polypropylene template induced orientation of fibrous nano- and micro-assemblies in the PANI macrostripe. The alignment of fibrous assemblies in the PANI stripe are characterized by SEM. With increasing draw ratio of the polyproplene template from 0% to 300%, the microscopic fibrous PANI assemblies became aligned from unordered arrangement. The alignment of the polymer chain and its crystalline in the polypropylene template was characterized by WAXS. With increasing draw ratio of the template, its scattering pattern became a discrete point from a dispersion ring. Scale bar corresponds to 20 μm in SEM images.

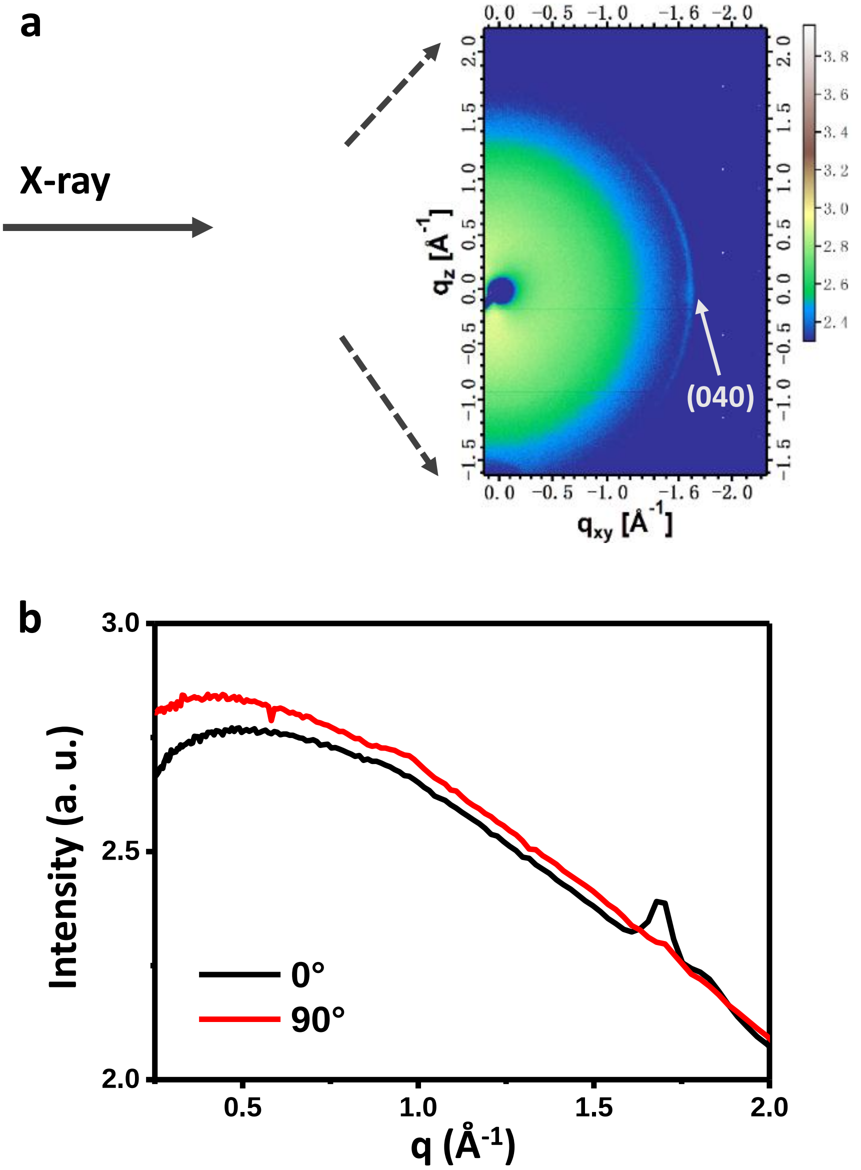

Supplementary Figure 2 | WAXS of the macro stripe. a, 2D WAXS pattern of the PANI macrostripe. The independent dispersive scattering point focusing on the equatorial direction (0°) is attributed to the π-π stacking between PANI molecules, but it is absent in the meridian direction (90°) due to the alignment of the fibrous nano- and micro-assemblies. **b,** Integration of the 2D WAXS along the equatorial (0°) and meridian directions (90°). The scattering peak at 1.7 Å attributed to the π-π stacking between PANI molecules appeared only in the integration curve along the equatorial direction.

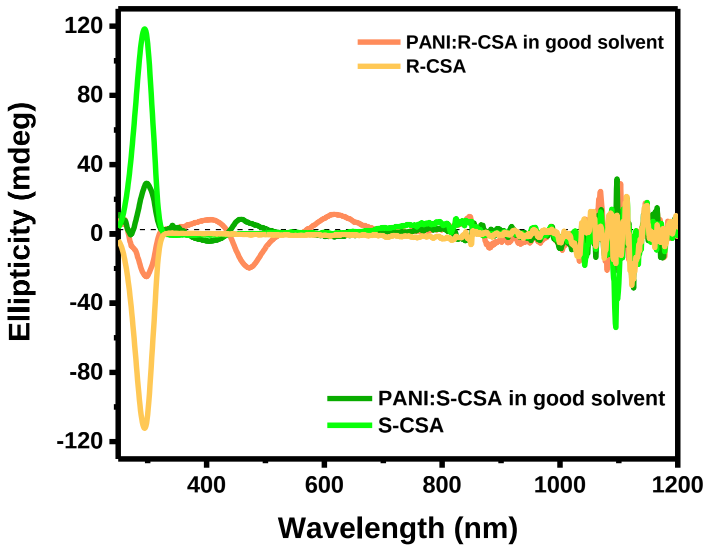

Supplementary Figure 3│The detailed CD the CSA and PANI:CSA that dissolved in methanol and $CHCl_3$/THF, respectively.

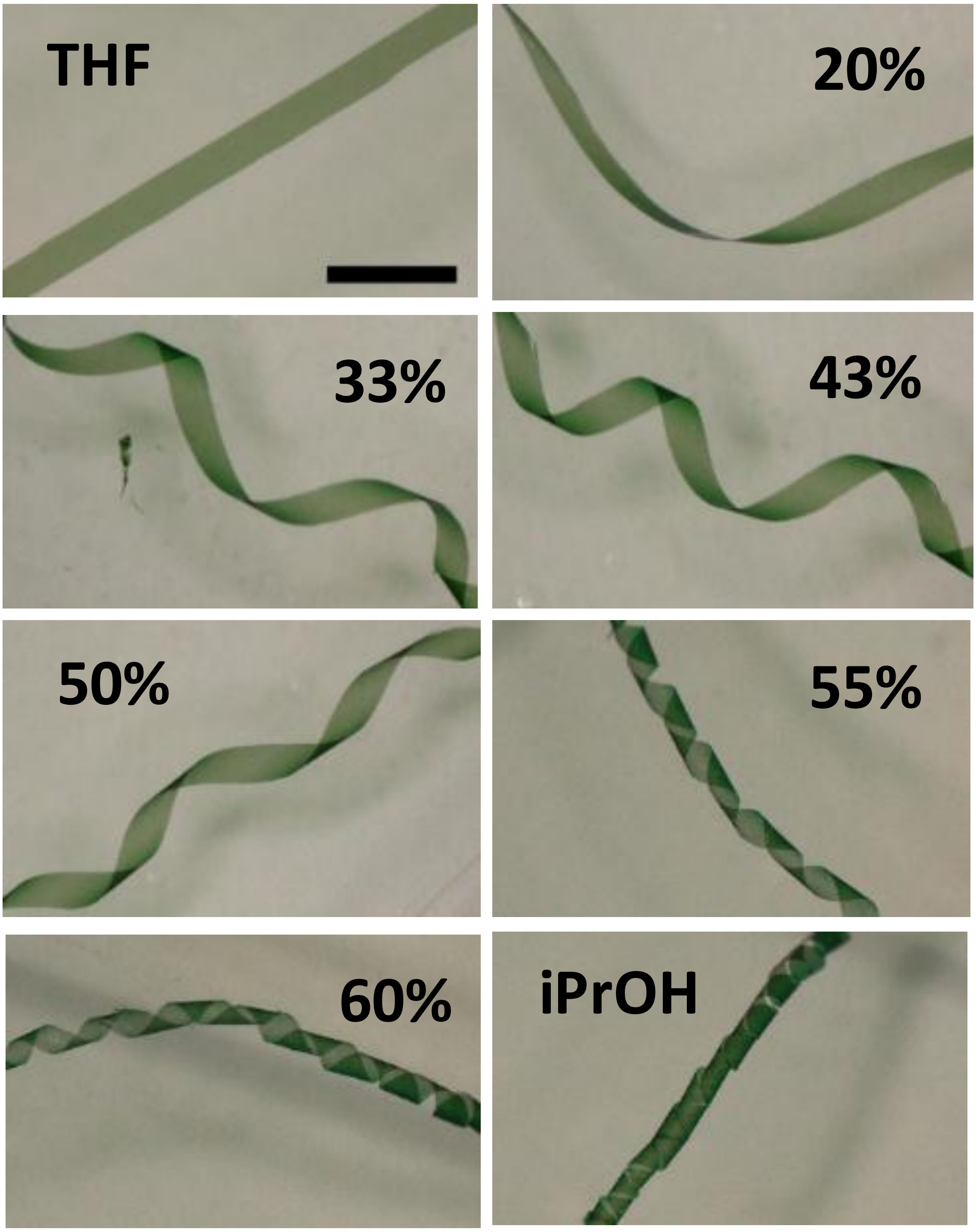

Supplementary Figure 4│Shape change of the PANI:*R*-CSA macrostripe with Φ_{iPrOH}. The photographs of the right-handed helical ribbon with different feature size of helicity under different Φ_{iPrOH}. By introducing iPrOH into THF, the flat macrostripe shrunk into right-handed helical ribbon. Scale bar corresponds to 1 mm.

S2. Supplementary Notes

Supplementary Note 1. Details about SAED

The SAED and corresponding TEM images of the macrostripe are displayed in Figure 1d. Diffused reflections (040) corresponding to *d*-spacing of approximately 3.9 Å resulted from the π-plane of PANI molecules stacked face-to-face along the long axis of the microfiber in the stripe. Sharp reflections (100) corresponding to *d*-spacing of *ca.* 6.1 Å originated from the arrangement of CSA molecules between neighboring PANI chains.[1,2]

Supplementary Note 2. Details about CD and g-factor

In this study, circular dichroism (CD) signals of CSA and CSA-doped PANI (PANI:CSA) appeared at *ca.* 300 and corresponds to their absorption peaks at 300 nm in the ultraviolet-visible-near-infrared (UV-vis-NIR) spectrum. CD bands at high wavelengths of PANI:CSA are relatively weak (enlarged image in Supplementary Fig. 3). CD signals of the macroribbons appeared at *ca*. 360 and 470 nm, which correspond to the absorption peaks at *ca.* 355 and 470 nm in the UV-vis-NIR spectrum, respectively. The bisignate Cotton effect bands of the macroribbons in CD spectra that appeared at 650 and 1000 nm correspond to the main absorption peaks at *ca.* 830 nm in the UV-vis-NIR spectrum. CD bands at 360 and 470 nm are associated with the low wavelength polaron and the π-π* absorption band at 355 and 470 nm, whereas the bisignate Cotton effect bands at 650 and 1000 nm are associated with the high wavelength polaron absorption band at ca. 830 nm in the UV-vis-NIR spectrum (see CD and UV-vis-NIR spectra in Figs. 1, 2, and 3). The CD band at 360 nm is attributed to the CSA incorporated in the PANI polymer, whereas the band at 470 nm is attributed to the optical activity in the PANI polymer backbone deriving from its helical chain configuration.[3] The bisignate CD bands at 650 and 1000 nm are attributed to the supramolecular chirality derived from the chiral exciton coupling of the interchain helical stacking of PANI molecules.[4]

g-factor, also called anisotropy of dissymmetry factor, is a useful derived quantity from CD and its corresponding absorption. $g = \Delta\varepsilon/\varepsilon$, where g is the anisotropy factor, *i.e.*, *g*-factor; $\Delta\varepsilon$ and ε are molar

CD and molar excitation, respectively. Given that CD and absorbance measurements are performed on one and the same sample, g-factor is independent of the sample concentration and of the optical path length. Thus, it is defined only as $\varepsilon\neq0$, which is in accordance with CD and absorption bands.[5]

The chiroptic features of PANI:CSA and its assemblies here are different from PANI:CSA aggregates prepared in aqueous-phase synthesis either by electrochemical[6] or chemical methods.[7] In detail, the remarkable difference is that the present PANI:CSA structures exhibiting an inverted sign for the character CD bands at ca. 450 nm comparing with its electrochemically synthesis and aqueous-phase synthesis analogous in previous reports. During the process of synthesis by electrochemical polymerization of aqueous aniline in the presence of enantiomeric CSA[6,8,9] or synthesis by chemical oxidization of aqueous aniline using ammonium persulfate as an oxidant in the presence of enantiomeric CSA,[10] the PANI aggregated during the polymerization in aqueous phase. They possess similar chiroptic features at ca. 450 nm. Whereas, in the present work, the PANI was synthesis by chemical oxidization of aniline in organic solvent using DDQ as an oxidant in the presence of enantiomeric CSA, then the as-prepared PANI was assembled slowly in organic solvent with high G/P ratio. This PANI:CAS solution and its assemblies possesses similar chiroptic features with emeraldine base doped by CSA in organic solutions.[11,12] The difference in their chiroptical feature at long wavelengths region may result from the in-situ doping or secondary doping of PANI by CSA.

Supplementary Note 3. Density functional theory calculation

The geometries of PANI molecule with eight repeating units were first optimized under b3lyp/6-31g** level. The supramolecular complexes of PANI: *S*-CSA and PANI: *R*-CSA were built based on the theoretical and experimental results.[2] Supramolecular structures of PANI: *S*-CSA and PANI: *R*-CSA were optimized with long-range corrected functional cam-b3lyp and 6-31g** basis set. Subsequently, dimer complexes of PANI: *S*-CSA and PANI: *R*-CSA were built based on the experimental SAED and WAXS data and further optimized under cam-b3lyp/6-31g** level. To simulate the environments of iPrOH and THF solutions, a polarized continuum model (PCM) was used (Supplementary Fig. 5).

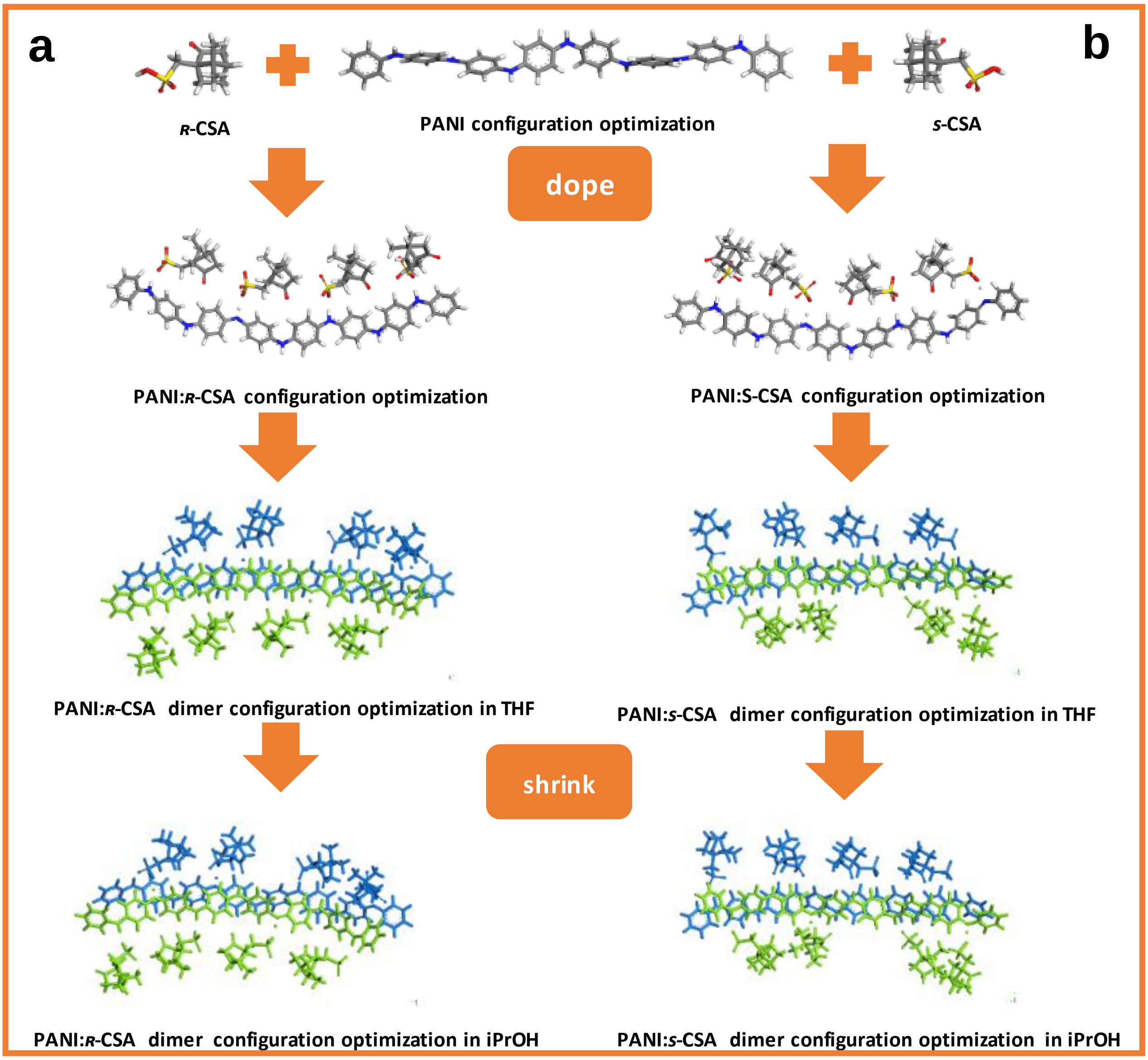

Supplementary Figure 5│Molecular and supramolecular structures doped by *s*-CSA and *R*-CSA optimized with long-range corrected functional cam-b3lyp and 6-31g basis set, respectively. a,** *R*-CSA and PANI: *R*-CSA, dimer complexes of PANI:*R*-CSA and **b,** *s*-CSA, PANI:*s*-CSA, dimer complexes of PANI:*s*-CSA in simulative environment of THF and iPrOH optimized under cam-b3lyp/6-31g** level, respectively. Middle, the PANI molecule with eight repeating units optimized under b3lyp/6-31g** level.

Based on the aggregation structure of the dimer, the π-π *d*-spacing between the PANI molecules was calculated using

$$\bar{d} = \frac{1}{n}\sum_{k=1}^{n} d_k^{\pi\text{-}\pi}, \tag{1}$$

where $d^{\pi\text{-}\pi}$ is the distance between π planes of two approximately parallel stacked benzene rings, as shown in Supplementary Figure 5, calculated as

$$d^{\pi\text{-}\pi} = \frac{1}{2}\left(d_{ij} + d_{ji}\right) = \frac{1}{2}\left(\boldsymbol{n}_i \cdot \boldsymbol{r}_{ij} + \boldsymbol{n}_j \cdot \boldsymbol{r}_{ji}\right), \tag{2}$$

where $\boldsymbol{r}_{ij} = \boldsymbol{x}_j^0 - \boldsymbol{x}_i^0$ and $\boldsymbol{r}_{ji} = \boldsymbol{x}_i^0 - \boldsymbol{x}_j^0$ are vectors between the centers of the two benzene rings, *i.e.,* $\boldsymbol{x}_i^0$ and $\boldsymbol{x}_j^0$; $\boldsymbol{n}_i$ and $\boldsymbol{n}_j$ are the normalized normal vectors of the corresponding π planes, respectively, and obtained by fitting the coordinates of the six atoms into a plane as follows:

$$\boldsymbol{n} \cdot \left(\boldsymbol{x} - \boldsymbol{x}^0\right) = 0. \tag{3}$$

Accordingly, the intermolecular distances between the PANI: *s*-CSA and PANI: *R*-CSA dimers in THF and iPrOH were calculated, respectively (Supplementary Table 1). The corresponding strain of the fibrous nano-assemblies in the PANI microfiber $\varepsilon_0^{\mathrm{f}}$ was calculated using

$$\varepsilon_0^{\mathrm{f}} = \frac{\bar{d}_{\mathrm{iPrOH}} - \bar{d}_{\mathrm{THF}}}{\bar{d}_{\mathrm{THF}}}. \tag{4}$$

The change of *d*-spacing of intermolecular π-π stacking of the stripe was further verified by WAXS. The stripes immerged in the solvents of THF and iPrOH were detected in quartz capillaries, respectively (Supplementary Fig. 7). The *d*-spacing of intermolecular π-π stacking of the stripe immerged in the THF and iPrOH are *ca*. 3.468Å and 3.436Å, respectively. And their difference value is *ca*. 0.032 Å. According to the DFT calculation, it is *ca*. 0.03 Å. The distance change of intermolecular *d*-spacing of π-π stacking between theoretical and experimental values, as well as their percentage change are congruent. To get distinguishable scattering signals, we concentrated the stripes as thick as possible resulting in the scattering pattern with dispersion ring rather than separated points.

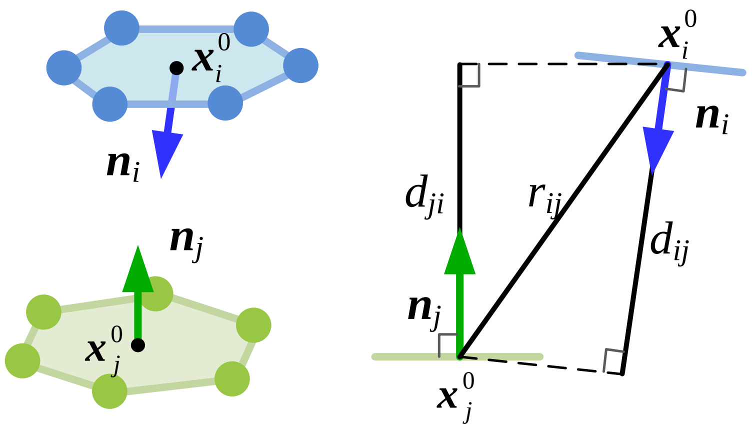

Supplementary Figure 6 | Schematic for calculating $d^{\pi\text{-}\pi}$. The model that was used to calculate the distance between π planes of the two approximately parallel stacked benzene rings in PANI:CSA dimer.

Solvents / Dopants	The average $d^{\pi\text{-}\pi}$ (Å)		Strain $\varepsilon_0^{\mathrm{f}}$
	THF	iPrOH	
R-CSA	3.91209	3.88095	-0.00796
S-CSA	3.89014	3.75772	-0.03404

Supplementary Table 1 | Calculated average distance between π planes of two approximately parallel stacked benzene rings $d^{\pi\text{-}\pi}$ of the PANI:S-CSA and PANI:R-CSA dimers in THF and iPrOH, and corresponding calculated strain of the fibrous nano-assemblies in the PANI microfiber $\varepsilon_0^{\mathrm{f}}$.

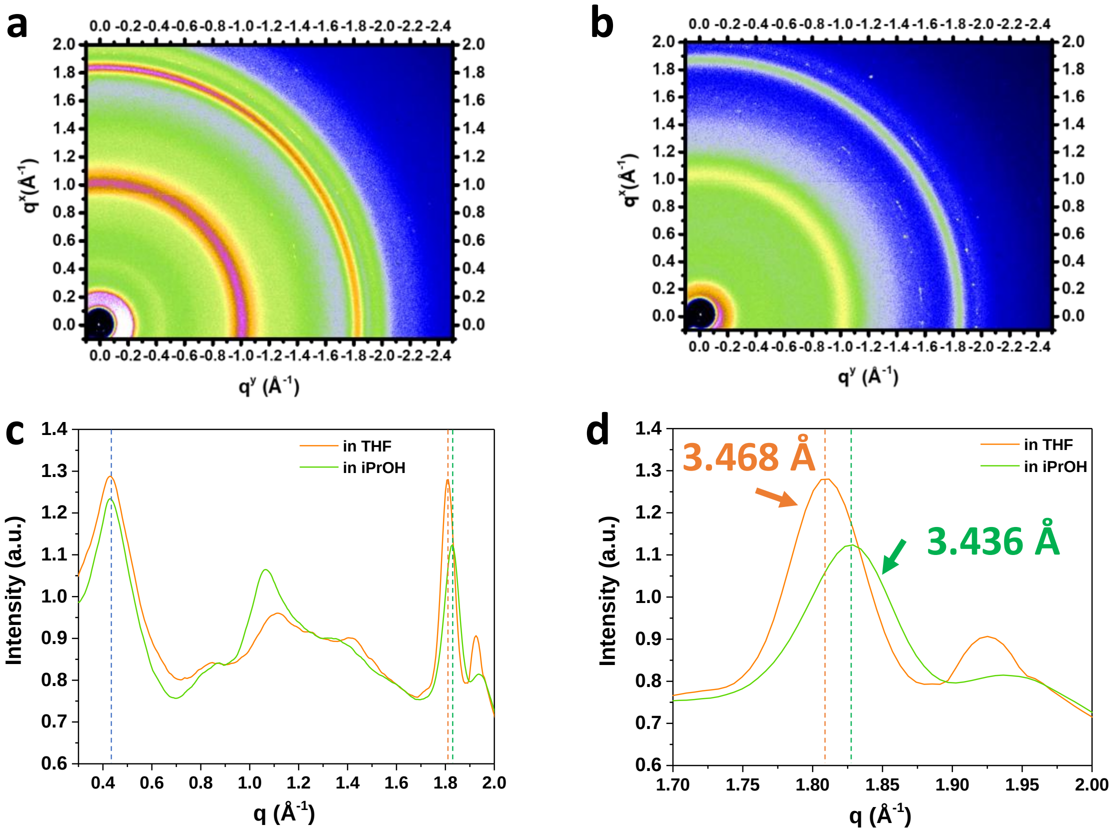

Supplementary Figure 7 | a, 2D WAXS of PANI:CSA emerged in THF; b, 2D WAXS of PANI:CSA emerged in iPrOH; c, Integral curves of corresponding 2D WAXS of PANI:CSA emerged in THF and iPrOH, respectively; d, The local enlarged integral curves from (c).

Supplementary Note 4. Multi-scale chemo-mechanical modeling and analysis

Supplementary Note 4.1. Microscale mechanical analysis

The spontaneous shrinkage of fibrous nano-assemblies in PANI microfibers induced axial strain, $\varepsilon_0^{\mathrm{f}}$, along their long axes. Given that the microscopic fibrous morphologies differ at the two encounter surfaces of the macroscopic stripe, projections of the axial strain along direction 1, $\varepsilon_{11}^{\mathrm{m}}$, varied (Fig. 4b). For the inside surface, both microscopic fiber and nano-assemblies are supposed to align parallel to direction 1 in the microscale. Therefore, the strains on these surfaces are considered as follows:

$$\varepsilon_{11}^{\mathrm{m-}} = \varepsilon_0^{\mathrm{f}}, \quad (5)$$

$$\varepsilon_{22}^{\mathrm{m-}} = \gamma_{12}^{\mathrm{m-}} = 0. \quad (6)$$

On the outside surface, the nano- and micro- assemblies are uniform single-handed helical with numerous interfaces (the junction where the helical microfibers bond with each other with different mechanical properties from that within the body of the fibers) between them; thus, the macroscopic strains should originate from the deformations of both microscopic fibers and interfaces between them. First, the shrinkage of the nano-assemblies induced deformation in the microscopic fibers. However, the direction of the shrinkage strain $\varepsilon_0^{\mathrm{f}}$ deviated from direction 1 due to the helical angle α of the fibrous nano-assemblies. The normal strain in direction *x* on the microscopic fiber is obtained via tensor transformations using

$$\varepsilon_{11}^{\mathrm{F}} = \varepsilon_0^{\mathrm{f}} \cos^2 \alpha, \quad (7)$$

where α is the average helical angle between the fibrous nano-assemblies and direction 1. Meanwhile, two strain components, *i.e.*, normal strain in direction 2 $\varepsilon_{22}^{\mathrm{F}}$ and shear strain γ_{12}^{F}, were derived on the helical microscopic fiber as follows:

$$\varepsilon_{22}^{\mathrm{F}} = \varepsilon_0^{\mathrm{f}} \sin^2 \alpha, \quad (8)$$

$$\gamma_{12}^{\mathrm{F}} = \varepsilon_0^{\mathrm{f}} \sin 2\alpha . \tag{9}$$

Second, the deformation of microscopic fibers transferred to the whole surface through the interfaces. Among three components of strain tensor, the strain in direction 1 $\varepsilon_{11}^{\text{m-}}$ is hardly affected by the weak interface, because both macroscopic fibers and the interfaces between them are parallel to direction 1. Therefore, $\varepsilon_{11}^{\text{m-}}$ is written as

$$\varepsilon_{11}^{\text{m-}} = \varepsilon_{11}^{\mathrm{F}} = \varepsilon_0^{\mathrm{f}} \cos^2 \alpha . \tag{10}$$

Transfer of normal strain in direction 2 and shear strain from the microscopic fibers to the macroscopic stripe may be restrained due to the failure of the interfaces. Therefore, the strain $\varepsilon_{22}^{\text{m-}}$ is set to 0 for simplicity, which indicates that $\varepsilon_{22}^{\mathrm{F}}$ could be counterbalanced by the deformation of the interfaces. For the shear strain of the macroscopic stripe, we considered the shear strain of the interface γ_{12}^{i} induced by the shear stress from microscopic fiber τ (Supplementary Fig. 7), which is expressed as

$$\tau = G^{\mathrm{F}} \gamma_{12}^{\mathrm{F}}, \tag{11}$$

where G^{F} is the shear modulus of the microfiber. As illustrated in Supplementary Figure 7, the shear strain of the surface is

$$\gamma_{12}^{\text{m-}} = \frac{\Delta}{W} = \frac{\Delta_{\mathrm{F}} - \Delta_{\mathrm{i}}}{W}, \tag{12}$$

where W is the width of the periodical unit cell. Δ_{F} and Δ_{i} are the shear displacements for the fiber and its interface, respectively, and expressed as

$$\Delta_{\mathrm{F}} = c_{\mathrm{F}} W \gamma_{12}^{\mathrm{F}}, \tag{13}$$

$$\Delta_{\mathrm{i}} = c_{\mathrm{i}} W \gamma_{12}^{\mathrm{i}} . \tag{14}$$

where c_{F} and c_{i} are the area fraction for fiber and interface, respectively, and satisfy $c_{\mathrm{F}} + c_{\mathrm{i}} = 1$. Therefore, the strain $\gamma_{12}^{\text{m-}}$ can be rewritten as

$$\gamma_{12}^{\text{m-}} = \gamma_{12}^{\text{F}}\left(1 - c_{\text{i}} - c_{\text{i}}\frac{\gamma_{12}^{\text{i}}}{\gamma_{12}^{\text{F}}}\right). \tag{15}$$

Denoting $\xi = 1 - c_{\text{i}} - c_{\text{i}}\,\gamma_{12}^{\text{i}}/\gamma_{12}^{\text{F}}$, the shear strain $\gamma_{12}^{\text{m-}}$ was then modified as

$$\gamma_{12}^{\text{m-}} = \xi\varepsilon_0^{\text{f}}\sin 2\alpha\,, \tag{16}$$

where ξ can be considered as a coefficient and satisfies $0 \le \xi \le 1$.

Suppose that the interface has a shear constitutive relation as

$$\tau^{\text{i}} = G^{\text{F}}A\left(\gamma_{12}^{\text{i}} + \sqrt{B\gamma_{12}^{\text{i}} + \left(\gamma_{12}^{\text{i}}\right)^2}\right), \tag{17}$$

the coefficient ξ is then expressed as

$$\xi = 1 - c_{\text{i}} - \frac{c_{\text{i}}\gamma_{12}^{\text{F}}}{A\left(AB + 2\gamma_{12}^{\text{F}}\right)}. \tag{18}$$

The area fraction of interface c_{i} is far less than 1, so Supplementary Equation 18 can be simplified as

$$\xi \approx 1 - \frac{c_{\text{i}}\gamma_{12}^{\text{F}}}{A\left(AB + 2\gamma_{12}^{\text{F}}\right)}. \tag{19}$$

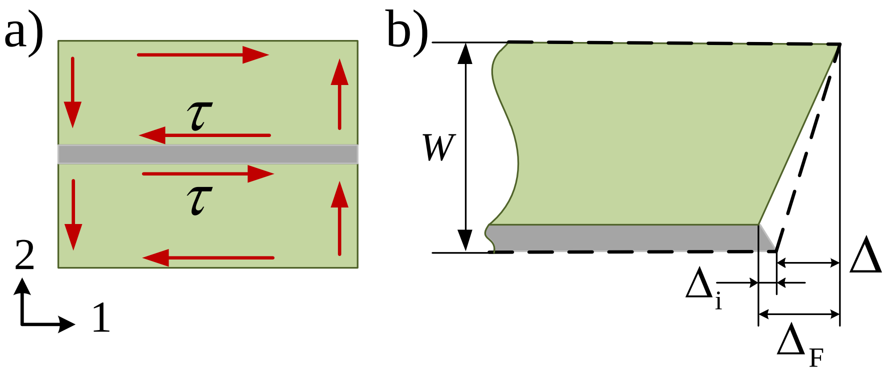

Supplementary Figure 8 | Schematic of the shear strain of the outside surface consisting of helical nano- and micro-assemblies. a, Shear stress originating from the spontaneous shear strain of the microfiber. **b,** Sketch of the shear strain composition.

Ultimately, the strain differences between the two surfaces is expressed as

$$\Delta\varepsilon_{11} = \varepsilon_{11}^{\text{m+}} - \varepsilon_{11}^{\text{m-}} = \varepsilon_0^{\text{f}} \cos^2\alpha - \varepsilon_0^{\text{f}} = -\varepsilon_0^{\text{f}} \sin^2\alpha , \tag{20}$$

$$\Delta\varepsilon_{22} = \varepsilon_{22}^{\text{m+}} - \varepsilon_{22}^{\text{m-}} = 0 , \tag{21}$$

$$\Delta\gamma_{12} = \gamma_{12}^{\text{m+}} - \gamma_{12}^{\text{m-}} = \xi\varepsilon_0^{\text{f}} \sin 2\alpha - 0 = \xi\varepsilon_0^{\text{f}} \sin 2\alpha . \tag{22}$$

Assuming that the spontaneous in-plane strain is distributed linearly along the direction of thickness, we can obtain the strain gradients along the thickness *h*, *i.e.,* the out-of-plane curvatures, as

$$\kappa_{11}^0 = \frac{\Delta\varepsilon_{11}}{h} = -\frac{\varepsilon_0^{\text{f}} \sin^2\alpha}{h} , \tag{23}$$

$$\kappa_{22}^0 = \frac{\Delta\varepsilon_{22}}{h} = 0 , \tag{24}$$

$$\kappa_{12}^0 = \frac{\Delta\gamma_{12}}{h} = \xi \frac{\varepsilon_0^{\text{f}} \sin 2\alpha}{h} . \tag{25}$$

where κ_{11}^0 and κ_{22}^0 are spontaneous bending curvatures in directions 1 and 2, and κ_{12}^0 is spontaneous bending curvature.

Supplementary Note 4.2. Macroscale mechanical analysis

To analyze out-of-plane deformation energy of the macroscopic helical ribbon, a cylindrical helical configuration possessing critical dimensions diameter *d* and helical pitch *p* is assumed first (Fig. 4c). In the global coordinate of *X*-*O*-*Y*, the ribbon exhibited a curvature of

$$\kappa_{XX} = \frac{2}{d} , \tag{26}$$

$$\kappa_{XY} = \kappa_{YY} = 0 . \tag{27}$$

In the local coordinate 1-*o*-2 on the ribbon, the curvature is decomposed into

$$\kappa_{11} = \kappa_{XX} \cos^2\theta , \tag{28}$$

$$\kappa_{22} = \kappa_{XX} \sin^2 \theta , \tag{29}$$

$$\kappa_{12} = \kappa_{XX} \sin 2\theta , \tag{30}$$

where θ is the pitch angle of the cylindrical helical ribbon using

$$\theta = \arctan\left({}^{p}\!/_{\pi d} \right) . \tag{31}$$

Treating the ribbon as a plastic thin plate with orthotropic stiffness, the out-of-plane deformation energy per unit area of the membrane is

$$E_{\text{out}} = \frac{1}{2} \mathbf{KDK}^{\mathrm{T}} , \tag{32}$$

where $\mathbf{K} = \left[\kappa_{11} - \kappa_{11}^0, \kappa_{22} - \kappa_{22}^0, \kappa_{12} - \kappa_{12}^0 \right]$ is the effective bending/torsional curvature, which can induce elastic energy; κ_{ij}^0 is the spontaneous bending/torsional curvature; and $\mathbf{D}$ is the out-of-plane stiffness matrix expressed as

$$\mathbf{D} = \frac{h^3}{12} \begin{bmatrix} \frac{E_1}{1-\nu_{12}\nu_{21}} & \frac{\nu_{21}E_2}{1-\nu_{12}\nu_{21}} & \\ \frac{\nu_{12}E_1}{1-\nu_{12}\nu_{21}} & \frac{E_2}{1-\nu_{12}\nu_{21}} & \\ & & G_{12} \end{bmatrix} , \tag{33}$$

where h is the thickness of the macrostripe; E_1 and E_2 are the effective elastic modulus of the two orthogonal directions 1 and 2, respectively; G_{12} is the in-plane shear modulus; ν_{12} and ν_{21} are Poisson's ratios, which satisfy

$$\nu_{21}E_2 = \nu_{12}E_1 . \tag{34}$$

Therefore, the energy of the macro stripe transformed into the cylindrical helical ribbon under the spontaneous bending (torsional) curvature is

$$E_{\mathrm{out}}=\frac{E_1h^3}{24(1-\nu_{12}\nu_{21})}\left[\begin{array}{l}\beta(1-\nu_{12}\nu_{21})\left(\kappa_{XX}\sin 2\theta-\kappa_{12}^0\right)^2\\+\left(\kappa_{XX}\cos^2\theta-\kappa_{11}^0\right)\left(\begin{array}{l}\nu_{12}\left(\kappa_{XX}\sin^2\theta-\kappa_{22}^0\right)\\+\kappa_{XX}\cos^2\theta-\kappa_{11}^0\end{array}\right)\\+\left(\kappa_{XX}\sin^2\theta-\kappa_{22}^0\right)\left(\begin{array}{l}\nu_{12}\left(\kappa_{XX}\cos^2\theta-\kappa_{11}^0\right)\\+\nu_{12}/\nu_{21}\left(\kappa_{XX}\sin^2\theta-\kappa_{22}^0\right)\end{array}\right)\end{array}\right], \tag{35}$$

where $\beta=G/E_1$ is the ratio of shear modulus to Young's modulus in direction 1.

Apparently, E_{out} is the function of cylindrical curvature κ_{XX} and helix angle θ. In the principle of minimum potential energy, under the condition of $\left(\kappa_{11}^0,\kappa_{22}^0,\kappa_{12}^0\right)$, the energy E_{out} should satisfy the set of equations below

$$\frac{\partial E_{\mathrm{out}}}{\partial \kappa_{XX}}=0 \text{ and } \frac{\partial E_{\mathrm{out}}}{\partial \theta}=0. \tag{36}$$

After solving the two equations above simultaneously, the corresponding cylindrical curvature κ_{XX} and helix angle θ are obtained, which were equivalent to the diameter d and pitch ratio p/d (Supplementary Equations 26 and 31; Figs. 3b and 4c).

Supplementary Note 4.3. Discussion on Multi-scale chemo-mechanical modeling

Substituting $\left(\kappa_{11}^0,\kappa_{22}^0,\kappa_{12}^0\right)$ in Supplementary Equations 23-25 into Supplementary Equation 35, the total energy is rewritten as

$$E_{\mathrm{out}}=\frac{E_1h\left(\varepsilon_0^{\mathrm{f}}\right)^2}{24(1-\nu_{12}\nu_{21})}\left[\begin{array}{l}\beta(1-\nu_{12}\nu_{21})\left(\lambda^{-1}\sin 2\theta+\xi\sin 2\alpha\right)^2\\+\left(\lambda^{-1}\cos^2\theta-\sin^2\alpha\right)\left(\nu_{12}\lambda^{-1}\sin^2\theta+\lambda^{-1}\cos^2\theta-\sin^2\alpha\right)\\+\lambda^{-1}\sin^2\theta\left(\nu_{12}\left(\lambda^{-1}\cos^2\theta-\sin^2\alpha\right)+\nu_{12}/\nu_{21}\,\lambda^{-1}\sin^2\theta\right)\end{array}\right], \tag{37}$$

where λ is a dimensionless parameter and expressed as

$$\lambda=-\varepsilon_0^{\mathrm{f}}/\left(\kappa_{XX}h\right). \tag{38}$$

By minimizing E_{out} with respect to λ and θ, we obtain the shape parameters of the helical ribbon. Supplementary Equation 37 also shows that λ and θ are only determined by five dimensionless parameters α, β, ν_{12}, ν_{21}, and ξ.

In Supplementary Equations 26 and 38, the diameter of the helical ribbon is computed as follows:

$$d = \frac{2\lambda h}{-\varepsilon_0^{\mathrm{f}}}, \quad (39)$$

which is proportional to the parameter λ.

To investigate how the parameters α, β, ν_{12}, ν_{21}, and ξ influenced the shape parameters λ and θ, these parameters were set as the calibrated values α =25°, β =1/4, ν_{12} =1/160, and ν_{21} =1/4 obtained from the experiments while one of them varying. The parameter ξ in (a), (b), (c) and (d) of Supplementary Figures 9 was set as 1. First, the effect of α is shown in Supplementary Figure 9a. The helical direction of the fibrous nano-assemblies (*i.e.,* the sign of the angle α) do not influence the diameter of helical ribbon (*i.e.,* λ) but determines the helical direction of the macroribbon (*i.e.,* the sign of the angle θ). A large α can induce small diameter *d* and angle θ. Increasing shear modulus ($G = \beta E_1$) could decrease helix diameter and increase the helix angle (Supplementary Fig. 9b). Increasing ν_{21} or decreasing ν_{12} could make the modulus in direction 2, E_2, soft, which results in a small diameter and large helical angle (black lines Fig. 3b). With the decrease in the coefficient ξ, the effect of spontaneous torsional curvature is suppressed gradually, which increases the diameter and reduces the helical angle.

Suppose that the shrinkage strain of nano-assemblies $\varepsilon_0^{\mathrm{f}}$ is proportional to the volume fraction of iPrOH Φ_{iPrOH} using

$$\varepsilon_0^{\mathrm{f}} = k_\varepsilon \Phi_{\mathrm{iPrOH}}, \quad (40)$$

according to Supplementary Equations 9, 19, and 40, the coefficient ξ is calibrated from the

experimental results as follows:

$$\xi = \frac{0.4}{0.4 + \Phi_{\text{iPrOH}}}. \tag{41}$$

With the increase in Φ_{iPrOH}, the deformation of the ribbon grew, and the load-transfer ability of the weak interface decreased. Therefore, the parameter λ increased and the helical angle θ decreased as Φ_{iPrOH} increased.

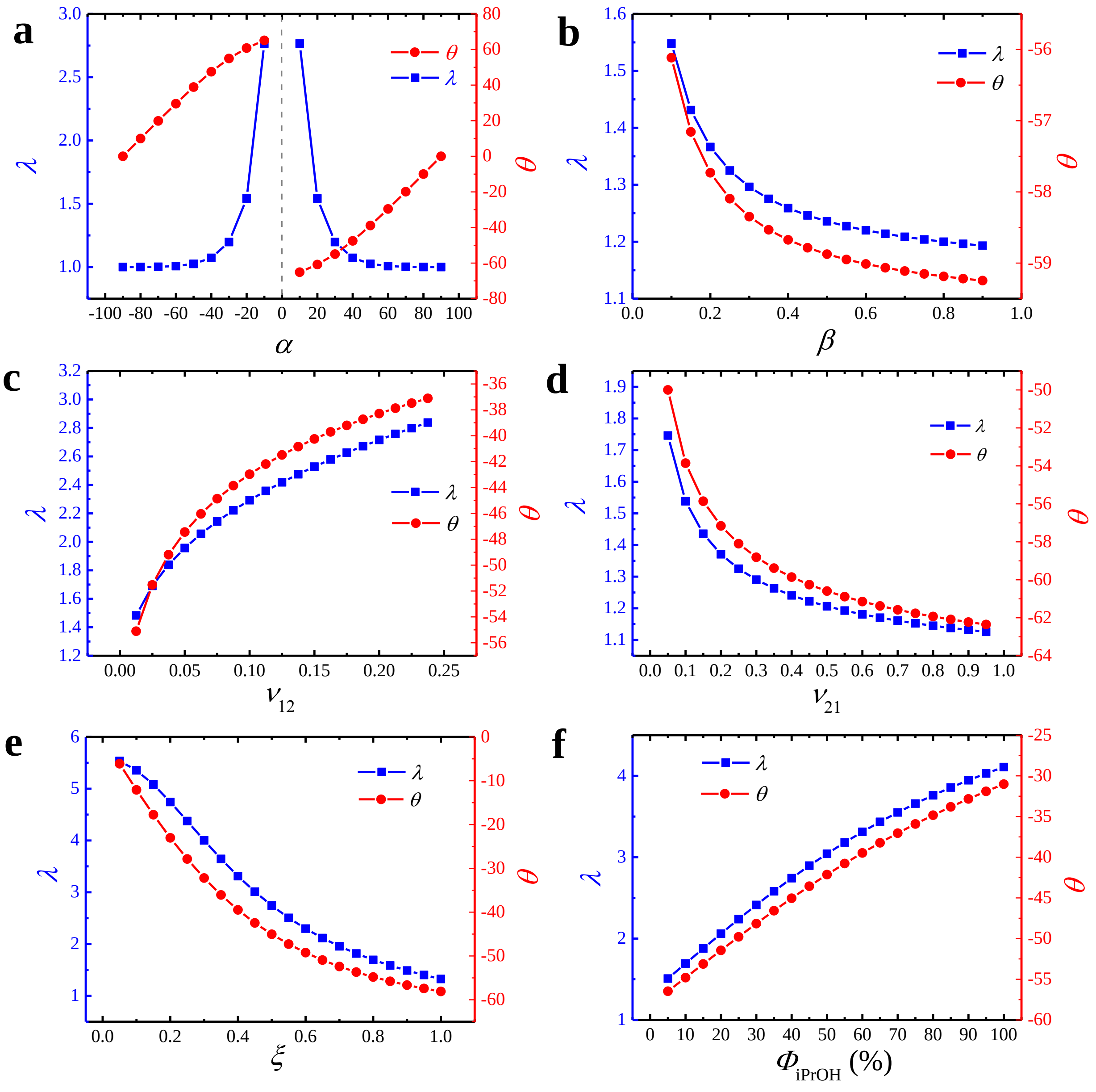

Supplementary Figure 9 | Curves of the shape parameters λ and θ of the helical ribbon for different a, The helical angle between fibrous nano-assemblies and direction 1 in the microfiber α ; **b,** The ratio of shear modulus to Young's modulus in direction 1 β ; **c and d,** Poisson's ratio ν_{12} and ν_{21} ; **e,** The coefficient for spontaneous torsional curvature ξ ; and **f,** Volume fraction of iPrOH Φ_{iPrOH} .

Supplementary Note 5. Explanations for majority rules experiments

Supplementary Note 5.1. Helicity of a single macroribbon with ee% of *s*-CSA deviates from ± 1

The analysis in Supplementary Note 4 is performed when the ee% of *s*-CSA of the system is fixed at 1 or -1, in which the chirality of the stripe keeps consistent with the molecular chirality of enantiomeric CSA-dopants. However, when ee% of *s*-CSA deviates from 1 or -1, the helicity of the macroribbon is determined by the ee% of *s*-CSA. We observed the microscopic morphologies of the ribbons doped by certain enantiomeric excess of *s*-CSA (ee% of *s*-CSA) by SEM (Supplementary Fig. 10). The net helicity of the helical locus in microscopic fibrous assemblies of the stripe was counted for each sample. According to statistics from the SEM images, the ee% of *s*-CSA has a nonlinear impact on the net helicity of the microscopic helical locus (Supplementary Fig. 11a).

Based on this observation, we construct the primary relationship between shape parameters of the macroribbon and its microscopic assemblies with a range of ee% of *s*-CSA. For an arbitrary single macroribbon, consider the volume (or area) fractions of helical locus in its microscopic assemblies with helical angles of $+\alpha$ and $-\alpha$ as c^+ and c^-, respectively, the net helicity of the helical locus in microscopic assemblies (χ) with helical angle of α can be written as

$$\chi = \frac{c^+ - c^-}{c^+ + c^-}. \qquad (42)$$

Assuming the helical locus with opposite helicity distributed randomly in the microscopic assemblies, thus the average strain on the microscopic fiber can be expressed as

$$\bar{\varepsilon} = \frac{1}{S}\int_S \varepsilon ds. \qquad (43)$$

According to Supplementary Equations 7-9 and Supplementary Equation 43 the average normal strains in direction 1 and 2 as well as shear strain of the micro assemblies on the outside surface are

$$\overline{\varepsilon_{11}^{\mathrm{F}}} = \varepsilon_0^{\mathrm{f}} \cos^2 \alpha, \qquad (44)$$

$$\overline{\varepsilon_{22}^{\mathrm{F}}} = \varepsilon_0^{\mathrm{f}} \sin^2 \alpha \,, \tag{45}$$

$$\overline{\gamma_{12}^{\mathrm{F}}} = \chi \varepsilon_0^{\mathrm{f}} \sin 2\alpha \,. \tag{46}$$

It can be found that only the shear strain $\overline{\gamma_{12}^{\mathrm{F}}}$ is affected by the sign of the helical angle α. Therefore, the spontaneous torsion curvature should be modified as

$$\kappa_{12}^{0} = \xi \frac{\chi \varepsilon_0^{\mathrm{f}} \sin 2\alpha}{h} \,, \tag{47}$$

and the coefficient ξ should be also modified according to its relationship to the average shear strain $\overline{\gamma_{12}^{\mathrm{F}}}$. According to Supplementary Equations 19, 40 and 41, it should be rewritten as

$$\xi = \frac{0.4}{0.4 + |\chi| \Phi_{\mathrm{iPrOH}}} \,. \tag{48}$$

Using the method developed in Supplementary Note 4.2, the pitch p, diameter d and the pitch ratio p/d of the helical macroribbon can be obtained (Supplementary Fig. 11c, d, and e). These parameters change according to variation of χ when Φ_{iPrOH} is fixed at 50%. With increase of c^+ or c^- (χ deviates from 0), the d decreases for the increasing spontaneous torsion curvature. The p/d is centrosymmetric about the origin of coordinates. From above, it can be found that the helicity of a given macroribbon (sign of the p/d) is determined by the helicity of the microscopic fibrous assemblies in the majority.

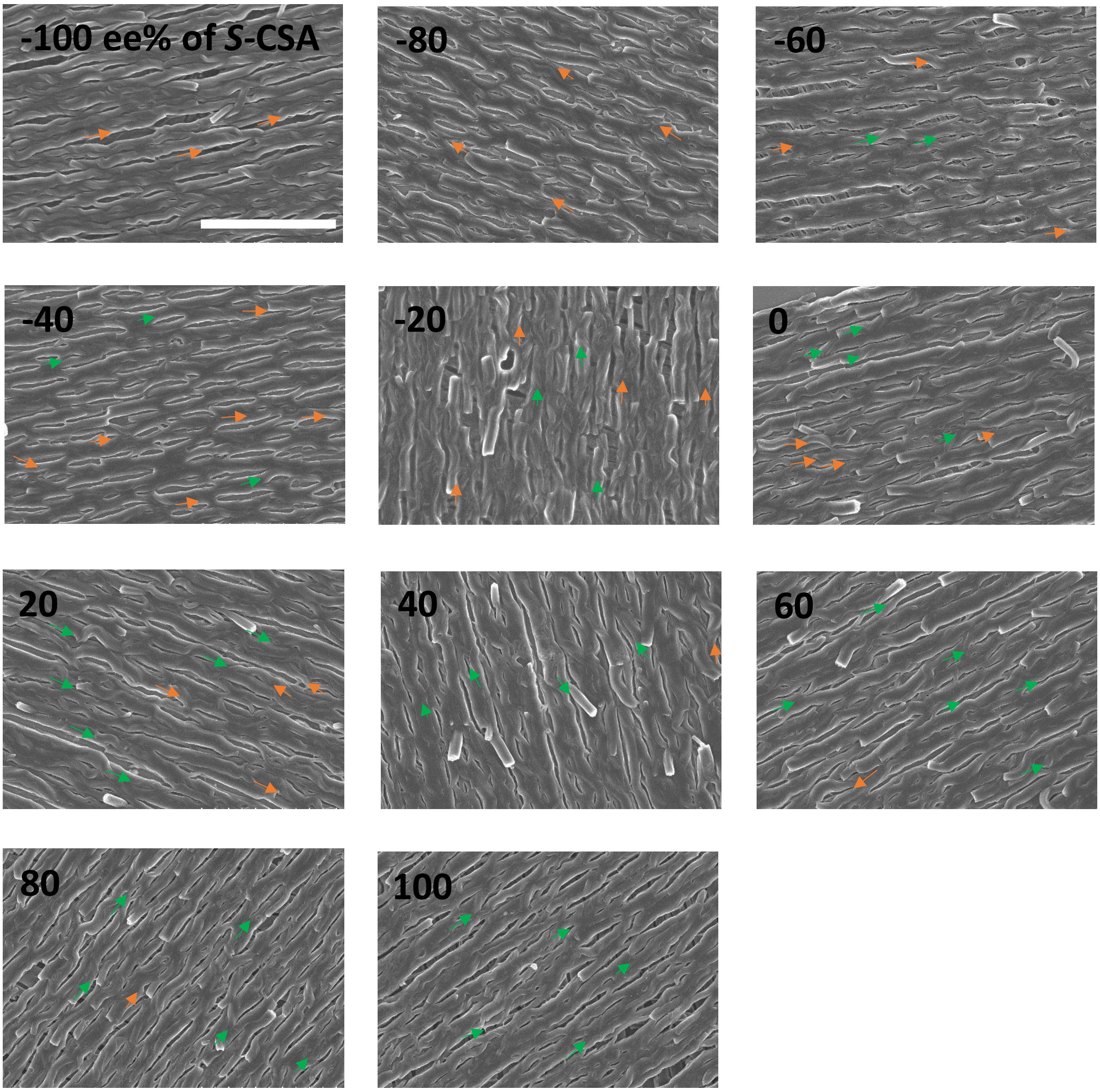

Supplementary Figure 10 | SEM images of the ribbons doped by CSA with various enantiomeric excess of *s*-CSA (ee% of *s*-CSA). The green and orange arrows indicate the left- and right-handed helical locus in the microscopic assemblies, respectively. Scale bar corresponds to 10 μm.

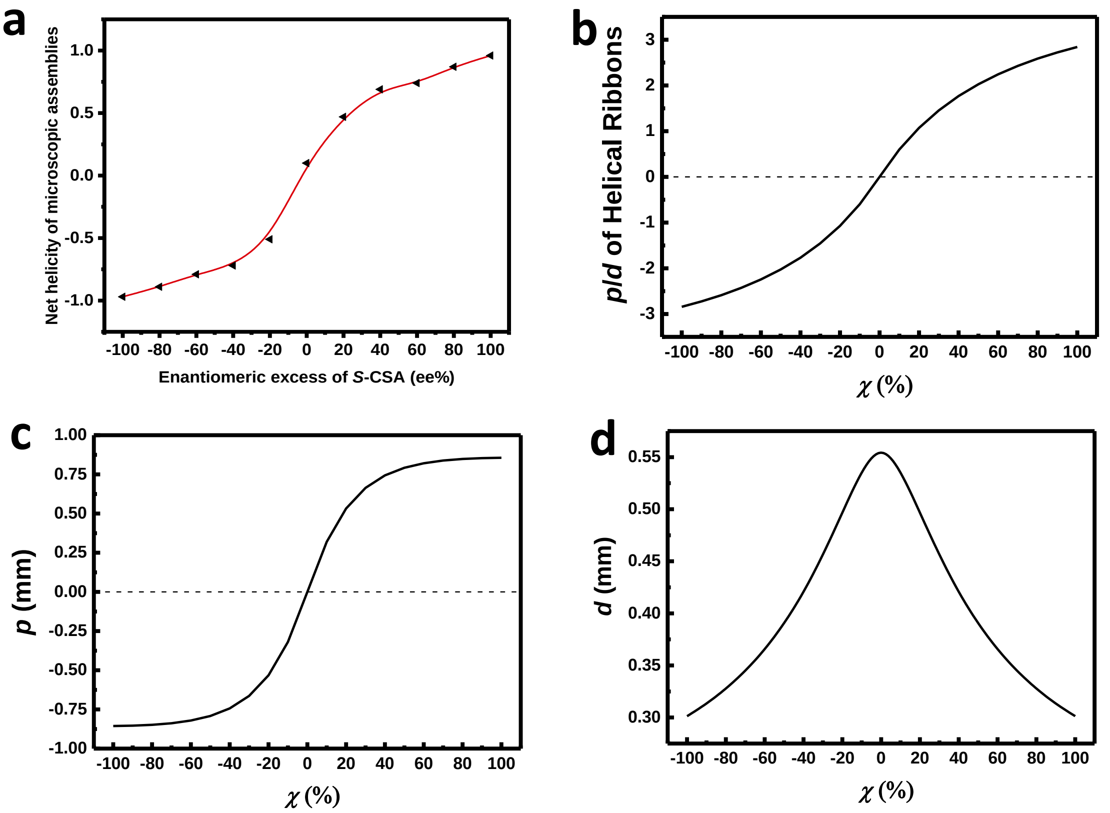

Supplementary Figure 11 | a, The disproportionate relationship between net helicity of the microscopic assemblies and enantiomeric excesses of *s*-CSA (ee%). **b, c, and d**, Calculated values of the pitch ratio *p/d*, the helical pitch *p* and the diameter *d* of the helical ribbon with various χ when Φ_{iPrOH} is set as 50%.

Supplementary Note 5.2. Chirality amplification in the majority rules experiments

In the majority rules experiments, when ee% of *s*-CSA deviates from 1 or -1, left- and right-handed macroscopic helical ribbons may appear simultaneously in a group of samples from a same batch, and the relationship between net helicity of the macroribbons and the ee% of *s*-CSA is nonlinear. From bottom to top, the helicity of the superior level is induced by the helicity of most of its subordinate. We denote the net helicity in the level of the supramolecular, microscopic and macroscopic as φ_{superm}, φ_{micro} and φ_{macro}, respectively, and assume they satisfy with the following relations

$$\varphi_{\text{superm}} = F_1(\text{ee\%}), \tag{49}$$

$$\varphi_{\text{micro}} = F_2(\varphi_{\text{superm}}), \tag{50}$$

$$\varphi_{\text{macro}} = F_3(\varphi_{\text{micro}}), \tag{51}$$

where the functions F_1, F_2 and F_3 describe the relation between the net helicity of the two adjacent levels, respectively.

These three functions can be molded as the Ball-box Matrix in probability theory[13-16]. Consider N subordinate helical assemblies (with opposite helicity denoted as l and r, respectively) to be equally assigned into M superior assemblies (with opposite helicity denoted as L and R, respectively), we mark the superior assemblies with more l-subordinates as L-superior and mark the superior assembly with more r-subordinates as R-superior. When the amounts of l-subordinate and r-subordinate are equal, the superior assembly loses its helicity theoretically, which is yet hard to reach experimentally. Here both N and M are large enough. We can define the net helidity of l-subordinate as

$$\psi = \frac{N_l - N_r}{N_l + N_r}, \tag{52}$$

where N_l and N_r are the amounts of l-subordinate and r-subordinate, respectively, and satisfy $N_l + N_r = N$. Similarly, we define the net helicity of L-superior as

$$\zeta = \frac{M_L - M_R}{M_L + M_R}, \tag{53}$$

where M_L and M_R are the amounts of L-superior and R-superior, respectively, and satisfy $M_L + M_R = M$. Then it can be found the relation between ψ and ζ is the function F_1, F_2 or F_3.

For superior assembly i, its helicity is determined by the net helicity of its subordinate assemblies (ψ_i). For all the superior assemblies, its net helicity (ζ) is influenced by the distribution of ψ_i, which should satisfy

$$\psi = \frac{1}{M}\sum_{i=1}^{M}\psi_i \, , \tag{54}$$

To probe the relation between ψ and ζ , we assume three distributions of ψ_i including two extreme boundaries:

(1) Step boundary: The subordinate assemblies with opposite helicity distribute absolutely uniform in each superior assembly proportionally, *i.e.* $\psi_i = \psi$ in every superior assembly. In this way, if ψ >0, all the superior assemblies will be *L*-superior; if ψ <0, all the superior assemblies will be *R*-superior. Therefore, this boundary relationship between ψ and ζ can be expressed as

$$\zeta_1 = \begin{cases} -1 & \psi < 0 \\ 0 & \psi = 0 \\ 1 & \psi > 0 \end{cases} . \tag{55}$$

(2) Linear boundary: Assuming that 'thoroughly chirality self-sorting' occurred in the subordinate level. The heterochiral subordinate assemblies cannot coexist in a same superior assembly, and the superior assemblies split according to the helicity of their subordinates. In this way, all the subordinates in a given superior assembly are left-handed or right-handed, *i.e.* the value of ψ_i is either 1 or -1. Denoting this bound as ζ_2, it should be equal to ψ , as

$$\zeta_2 = \psi \, . \tag{56}$$

(3) Continuous nonlinear relation: Generally, the situation is between the two extreme boundaries. The homochiral subordinates may develop into localized aggregations randomly. Accordingly, we suppose the subordinates with same helicity form homochiral intermediate aggregations, and a given superior assembly is consisted of *k* intermediate aggregations. Then the probability of a given superior assembly exhibiting left-handedness is P_L, in which over half of the intermediate aggregations are left-handed. The probability can be calculated by using a *k*-fold Bernoulli trial model, as

$$P_L = \sum_{i=\frac{k}{2}+1}^{k}\left(\binom{k}{i} p^i (1-p)^{k-i}\right) + \frac{1}{2}\left(\binom{k}{k/2} p^{k/2} (1-p)^{k/2}\right), \tag{57}$$

where $\binom{k}{i}=\frac{k!}{i!(k-i)!}$ is the binomial coefficient, and p is the probability of left-handed intermediate aggregation in one trial and satisfies $p=(\psi+1)/2$. It is noted that when the amount of *l*-subordinate and *r*-subordinate are equal (ψ_i=0), the superior assembly loses its helicity theoretically. However, exact ψ_i=0 is hardly to reach experimentally, thus we identified the probability with the assumption that the amount of *L*- and *R*-superior are equal in this case (as the second item in Supplementary Equation 57). Here *k* is assumed to be an even number to guarantee the existence of ψ_i =0 mathematically. According to probability theory[13], the fraction of *L*-superior is equal to P_L, thus the net helicity estimation ζ_3 can be derived as

$$\zeta_3 = 2P_L - 1. \qquad (58)$$

Supplementary Figure 12a present the function relation between ζ *a*nd ψ, *i.e.* the function F_i (*i*=1,2,3). ζ_1 and ζ_2 are the two extreme boundaries. The function relation between ζ_3 and ψ presents a sigmoid curve that locates between the two extreme boundaries, which represents the situations with "partially chiral self-sorting". It is also found that the slope at ψ =0 is dependent on the parameter *k*. The slope increases with increasing of parameter *k*. Especially, when *k*=2, ζ_3 degenerates into ζ_2, and when $k\to\infty$, ζ_3 degenerates into ζ_1. *k* can be regarded as the parameter that represents the nonlinear degree. The nonlinear degree increases with increasing of *k*. From the view of probability theory, this nonlinear phenomenon results from the distribution of helical units.

The relation between *g*-factor of supramolecular assemblies and ee% of *s*-CSA is nonlinear (Fig. 4f, the green line). However, the relation between net helicity of microscopic assemblies and *g*-factor of supramolecular assemblies as well as the relation between net helicity of macroscopic assemblies and net helicity of microscopic assemblies are closer to be linear (Supplementary Fig. 12b, c). Evidently, the chirality amplification occurred mainly in the process of supramolecular assemble.

Because F_i is always situated between the step and linear boundaries. And the relation between ee% and φ_{macro} are nested, which can be expressed as

$$\varphi_{\text{macro}} = F_3\left(F_2\left(F_1\left(\text{ee\%}\right)\right)\right). \tag{59}$$

As a result, once continuous nonlinear amplification occurred in a certain hierarchy, the relation between its subordinates and its superior level will always be sigmoid type. And the nonlinearity can be maintained or intensified but can be hardly degenerated in this situation. Therefore, the proportional relation between the net helicity of the helical locus in microscopic assemblies and ee% of *s*-CSA, as well as the relation between net helicity of the macroscopic assemblies and ee% of *s*-CSA are nonlinear with sigmoid type curves (Supplementary Fig. 11a, and the aurantia line in Fig. 4f).

So far, we have illuminated that the chirality amplification occurred in the stage of supramolecular assemble, and transfers to higher level by chirality induction. The chirality of enantiomeric CSA determines the helicity of the macroribbons by controlling the helical sense of the microscopic assemblies.

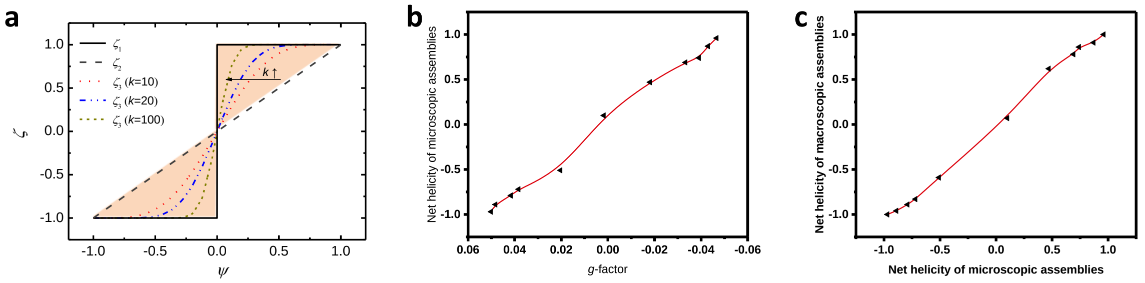

Supplementary Figure 12 | a, The function relation between ζ *and* ψ. The ζ_1 and ζ_2 are the two extreme boundaries represent the situations that with no self-sorting and with thorough chirality self-sorting, respectively. **b,** The linear relation between net helicity of microscopic assemblies and *g*-factor of supramolecular assemblies. **c,** The linear relation between net helicity of macroscopic assemblies and net helicity of microscopic assemblies.

Supplementary Note 6. Chirality perception mechanism of PANI:CSA stripes under stimuli of enantiomeric amines.

Upon adding of the chiral target species, the PANI stripes would de-dope and turn into blue from green, and its UV-vis-NIR spectra would blue shift accordingly (Supplementary Fig. 12a).[17] Therefore, we recorded the de-doping process of the stripe by UV-vis-NIR spectra upon adding a series of enantiomeric amines with different initial concentrations (examples see Supplementary Figs. 13 and 14).

We use attenuation of peak intensity at 470 nm of the PANI stripe with a given volume to demarcate its de-doping degree. The relationship between de-doping degree and time can be fitted well by the following exponent function:

$$I = \mathrm{e}^{-\omega t}. \tag{60}$$

where I is the normalized absorption intensity of the PANI stripe at 470 nm, and t is the de-doping time (Supplementary Fig. 13d, e). Derive I with respect to t, we obtain the de-doping rate r, as a function of time:

$$r = \frac{dI}{dt} = -\omega \mathrm{e}^{-\omega t}. \tag{61}$$

Where ω can be regarded as a parameter to judge the de-doping rate of different target species at given initial concentration c_0. The parameter ω is the function of c_0, and their relationship can be fitted well by a logarithmic function:

$$\omega = a + k_a \ln c_0. \tag{62}$$

Where k_a can be regarded as de-doping apparent rate constant of a specific target species (Supplementary Fig. 13b, c). For enantiomeric target species with chiral selectivity (2-aminohexane, 2-aminononane, 1-methyl-3-phenylpropylamine, 1-phenylbutylamine, and 2-methyl-1-phenylpropan-

1-amine), their constant k_a of target species with compatible chirality are an order of magnitude higher than that of with incompatible chirality ones (Fig. 5b, and Supplementary Table 2). Due to the difference in de-doping apparent rate constant k_a between chirality compatible and incompatible target species, the time for complete de-doping of PANI:CSA stripe induced by compatible target species are ca. 30 s to 60 s, whereas the time for complete de-doping of PANI:CSA stripe induced by incompatible target species are ca. 90 s to 150 s (Supplementary Fig. 12d, e). From the experimental observation, the point of distinct deformation of the PANI:CSA stripes occurred after adding of target species ca. 30 s to 50 s. Complete de-doping of PANI:CSA stripes induced by chiral compatible target species in advance of distinct deformation leads to regular helical deformation of the stripes, whereas complete de-doping of PANI:CSA stripes induced by chiral incompatible target species lag behind deformation leads to irregular deformation of the stripes.

Regarding the amino as the pivot of the target species, besides its configuration, both the substituent groups attached to its two sides influence its k_a. As volume of the substituent increases, k_a decreased. The (*s*)-(+)-2-aminobutane induces both PANI:S-CSA and PANI:R-CSA stripe turning into left-handed and right-handed helix without selectivity. Because of its easy-to-migrate small substituent groups, (*s*)-(+)-2-aminobutane leads to quick and complete de-doping in both PANI:*s*-CSA and PANI:*R*-CSA stripes, which results in no distinct difference for its k_a between PANI:*s*-CSA and PANI:*R*-CSA stripes. On the contrary, (1*s*)-2-(4-methylphenyl)-1-phenylethanamine induces irregular deformation in both PANI:*s*-CSA and PANI:*R*-CSA stripe without selectivity. Because of hindrance of aromatic substituents attached to both side of its the amino pivot, (1*s*)-2-(4-methylphenyl)-1-phenylethanamine is hard to induce complete de-doping in neither PANI:*s*-CSA nor PANI:*R*-CSA stripes. Low initial concentration c_0 of target species (below 5×10^{-4} μM at a given volume of PANI stripe) cannot induce the stripe de-doping completely, which leads to mild irregular deformation of stripes losing its chirality selectivity. This means the deformation behavior of the stripe is sensitive to the concentration.

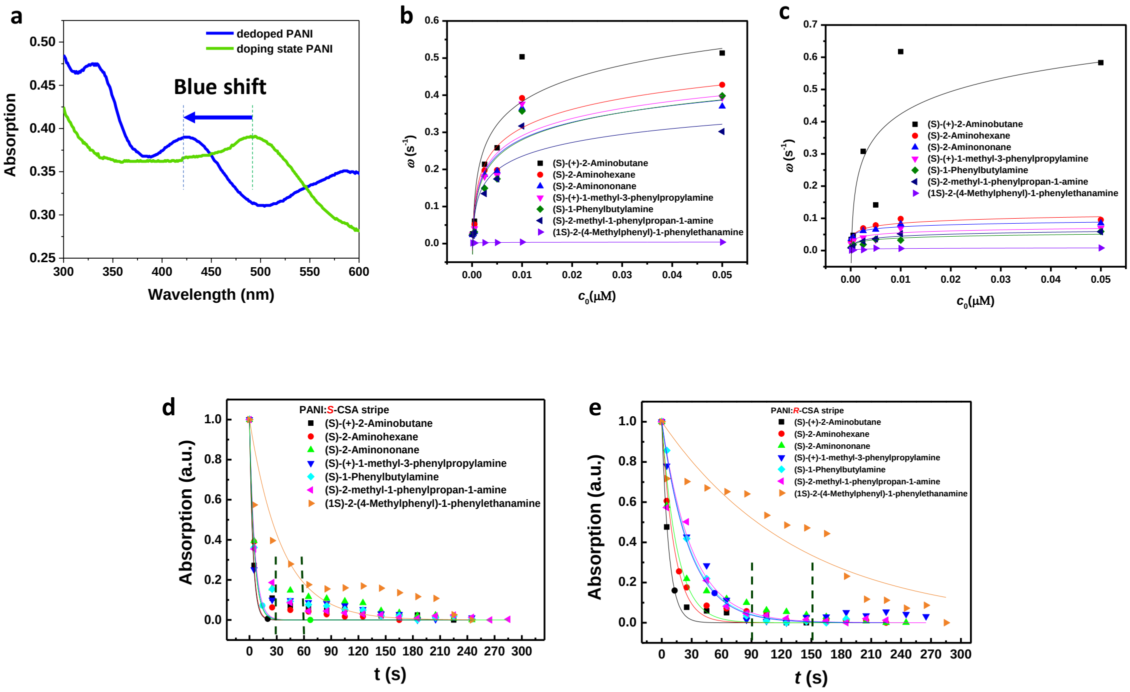

Supplementary Figure 13 | a, UV-vis-NIR spectrum of of PANI stripe blue shift according to its de-doping upon adding of of the chiral target species; **b**, **c**, Fitting the function relationship between parameter ω and c_0 by a logarithmic function; **d**, **e**, Example for the time difference of complete de-doping of PANI:*s*-CSA and PANI:*R*-CSA stripe by adding (*s*)-1-phenylbutylamine.

Target species / k ($s^{-1}\mu M^{-1}$)	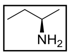 (S)-(+)-2-Aminobutane	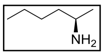 (S)-2-Aminohexane	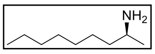 (S)-2-Aminononane	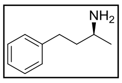 (S)-(+)-1-methyl-3-phenylpropylamine	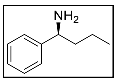 (S)-1-Phenylbutylamine	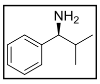 (S)-2-methyl-1-phenylpropan-1-amine	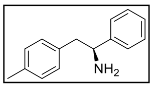 (1S)-2-(4-Methylphenyl)-1-phenylethanamine
PANI:*S*-CSA stripe	0.1005	0.0718	0.0683	0.0671	0.0648	0.0536	0.0004
PANI:*R*-CSA stripe	0.0892	0.0128	0.0091	0.0089	0.0082	0.0073	0.0013

Supplementary Table 2 | Constant k_a values of target species for PANI:CSA stripes with compatible and incompatible chirality.

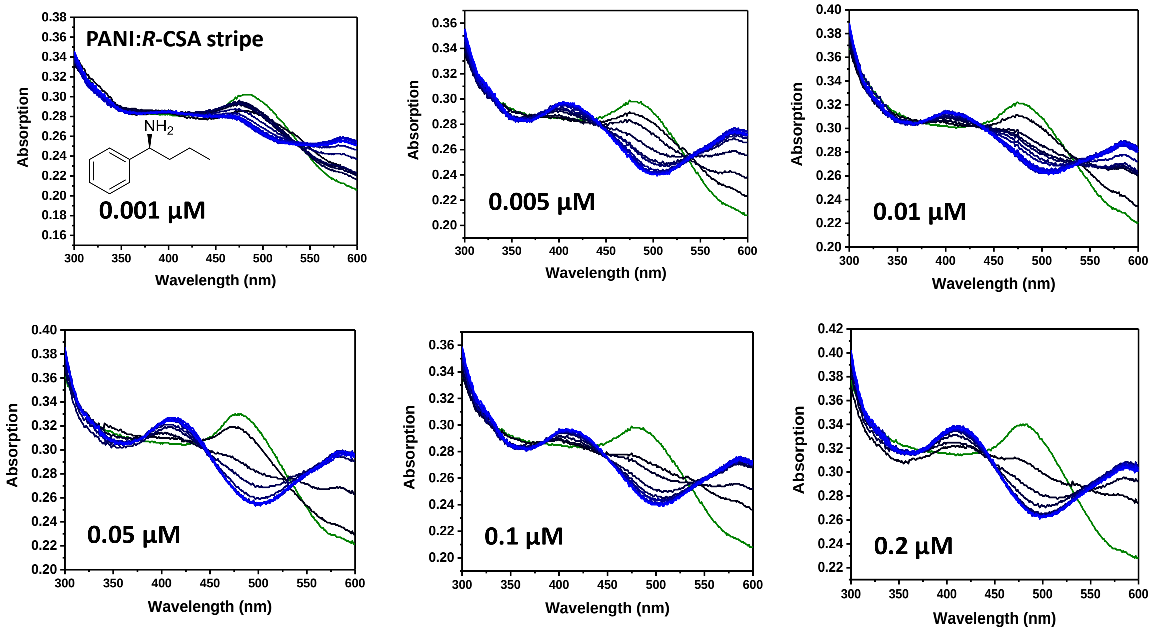

**Supplementary Figure 14 | ** Vis spectra of PANI:*R*-CSA stripe de-doping upon adding of a series of enantiomeric target species. Initial concentration of the target species is 0.05 µM.

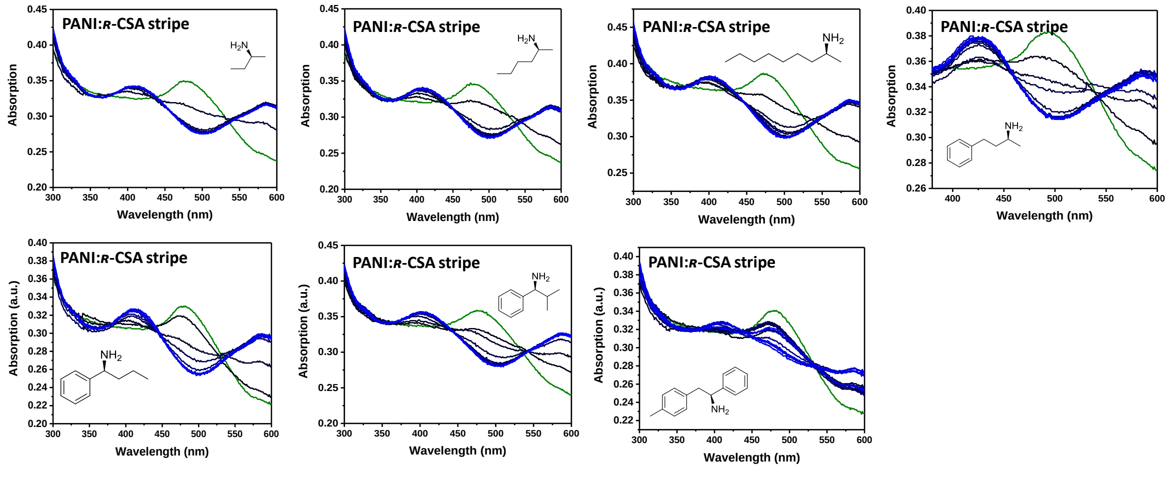

**Supplementary Figure 15 | ** Vis spectra of PANI:*R*-CSA stripe de-doping upon adding of (*S*)-1-Phenylbutylamine with a series of initial concentrations.

Supplementary Reference:

1 Yan, Y., Zhang, Y. J., Hu, W. P. & Wei, Z. X. Hierarchical Crystalline Superstructures of Conducting Polymers with Homohelicity. *Chem. Eur. J.* **16**, 8626-8630 (2010).

2 Zou, W. J. *et al.* Biomimetic Superhelical Conducting Microfibers with Homochirality for Enantioselective Sensing. *J. Am. Chem. Soc.* **136**, 578-581 (2014).

3 Kane-Maguire, L. A. P., MacDiarmid, A. G., Norris, I. D., Wallace, G. G. & Zheng, W. G. Facile preparation of optically active polyanilines via the in situ chemical oxidative polymerisation of aniline. *Synth. Met.* **106**, 171-176 (1999).

4 Kane-Maguire, L. A. & Wallace, G. G. Chiral conducting polymers. *Chem Soc Rev* **39**, 2545-2576 (2010).

5 Berova, N., Di Bari, L. & Pescitelli, G. Application of electronic circular dichroism in configurational and conformational analysis of organic compounds. *Chem Soc Rev* **36**, 914-931 (2007).

6 Majidi, M. R., Kanemaguire, L. A. P. & Wallace, G. G. Enantioselective Electropolymerization of Aniline in the Presence of (+)-Camphorsulfonate or (-)-Camphorsulfonate Ion - a Facile Route to Conducting Polymers with Preferred One-Screw-Sense Helicity. *Polymer* **35**, 3113-3115 (1994).

7 Yan, Y., Yu, Z., Huang, Y. W., Yuan, W. X. & Wei, Z. X. Helical polyaniline nanofibers induced by chiral dopants by a polymerization process. *Adv. Mater.* **19**, 3353-3357 (2007).

8 Li, W. G. & Wang, H. L. Electrochemical synthesis of optically active polyaniline films. *Adv. Funct. Mater.* **15**, 1793-1798 (2005).

9 Majidi, M. R., Kane-Maguire, L. A. P. & Wallace, G. G. Electrochemical synthesis of optically active polyanilines. *Aust J Chem* **51**, 23-30 (1998).

10 Yan, Y., Yu, Z., Huang, Y. W., Yuan, W. X. & Wei, Z. X. Helical polyaniline nanofibers induced by chiral dopants by a polymerization process. *Adv. Mater.* **19**, 3353-3357 (2007).

11 Havinga, E. E., Bouman, M. M., Meijer, E. W., Pomp, A. & Simenon, M. M. J. Large Induced Optical-Activity in the Conduction-Band of Polyaniline Doped with (1s)-(+)-10-Camphorsulfonic Acid. *Synth. Met.* **66**, 93-97 (1994).

12 Majidi, M. R., Kanemaguire, L. A. P. & Wallace, G. G. Chemical Generation of Optically-Active Polyaniline Via the Doping of Emeraldine Base with (+)-Camphorsulfonic or (-)-Camphorsulfonic Acid. *Polymer* **36**, 3597-3599 (1995).

13 Bertsekas, D. P. *Introduction to Probability: Dimitri P. Bertsekas and John N. Tsitsiklis*. (Athena Scientific, 2002).

14 van Gestel, J., van der Schoot, P. & Michels, M. A. J. Amplification of chirality in helical supramolecular polymers beyond the long-chain limit. *J. Chem. Phys.* **120**, 8253-8261 (2004).

15 van Gestel, J., van der Schoot, P. & Michels, M. A. J. Amplification of chirality in helical supramolecular polymers. *Macromolecules* **36**, 6668-6673 (2003).

16 Teramoto, A. Cooperative conformational transitions in linear macromolecules undergoing chiral

perturbations. *Prog. Polym. Sci.* **26**, 667-720 (2001).

17 Li, W. G. & Wang, H. L. Oligomer-assisted synthesis of chiral polyaniline nanofibers. *J. Am. Chem. Soc.* **126**, 2278-2279 (2004).
