# Supplementary material for: Macroscopic helical chirality and self-motion of hierarchical self-assemblies induced by enantiomeric small molecules
Source: Nat Commun. 2018 Sep 18;9:3808. doi: 10.1038/s41467-018-06239-5 (PMC6143534; doi:10.1038/s41467-018-06239-5)
Supplement: Supplementary file 3 — Description of Additional Supplementary Files [file 41467_2018_6239_MOESM3_ESM.pdf]

Description of Additional Supplementary Files

File Name: Supplementary Movie 1
Description: **Reversible shape changes between flat and helical of PANI:*s*-CSA macrostripe.** The PANI:*s*-CSA macrostripe curled into a left-handed helical ribbon when vast amounts of iPrOH were added into THF. By contrast, when the amount of THF dominated, the helical ribbon returned to its flat shape, and the process conducted in reverse. The video was sped up 10 times, to clarify the helical motion of the ribbon.

File Name: Supplementary Movie 1
Description: **Actuation behavior of PANI:*s*-CSA stripe when expose to mismatched chiral species.** When the PANI:*s*-CSA stripe exposed to (*R*)-(+)-2-aminohexane , slower de-doping with irregular locus happened in the stripe, which resulted in disordered deformation of the stripe. The video was sped up 10 times, to clarify the deformation characteristics of the ribbon.
